# Evidence-Based Practice Curriculum Development for Undergraduate Nursing Students: The Preliminary Results of an Action Research Study in Taiwan

**DOI:** 10.1097/jnr.0000000000000298

**Published:** 2019-07-16

**Authors:** Hsiao-Ying HUNG, Yu-Wen WANG, Jui-Ying FENG, Chi-Jane WANG, Esther Ching-Lan LIN, Ying-Ju CHANG

**Affiliations:** 1 MSN, RN, Doctoral Student, Department of Nursing, College of Medicine, National Cheng Kung University; 2 PhD, RN, Professor, Department of Nursing, College of Medicine, National Cheng Kung University; 3 PhD, RN, Associate Professor, Department of Nursing, College of Medicine, National Cheng Kung University; 4 PhD, RN, Professor, Institution of Allied Health Sciences and Department of Nursing, College of Medicine, National Cheng Kung University, and Director, Department of Nursing, National Cheng Kung University Hospital, College of Medicine, National Cheng Kung University.

**Keywords:** baccalaureate nursing education, curriculum design, evidence-based practice, teaching strategies, undergraduate nursing students

## Abstract

**Background:**

Equipping undergraduate nursing students with sufficient competence in evidence-based practice (EBP) is essential to meeting future practice needs. Integrating necessary EBP knowledge and skills systematically into the formal curriculum allows students to obtain better learning experience and outcomes. However, in Taiwan, a systematic nursing curriculum that integrates EBP concepts across the 4-year nursing baccalaureate program has not yet been developed. Moreover, engaging students in the clinical application of evidence remains a key challenge facing nursing education.

**Purpose:**

This study aimed to construct an EBP undergraduate nursing curriculum and develop clinical scenarios that support EBP teaching.

**Methods:**

Three cycles of action research, incorporating both focus group interviews and questionnaire surveys, were applied to construct and evaluate the appropriateness and feasibility of the EBP nursing curriculum and relevant teaching strategies.

**Results:**

An EBP nursing curriculum was constructed that integrates the three levels of learning objectives and corresponding learning outcomes, teaching content, and learning activities. Scenario activities were developed to familiarize students with the EBP process and to maximize their learning with regard to the clinical application of evidence. Next, a preliminary evaluation showed the appropriateness and feasibility of the developed curriculum, which was shown to foster the EBP competency of students and increase their confidence and positive attitudes toward EBP.

**Conclusions/Implications for Practice:**

A systematic EBP bachelor nursing curriculum with effective pedagogical strategies was developed. The associated process and the elicited information may offer a valuable reference for other nursing schools.

## Introduction

Evidence-based practice (EBP) is valued widely in clinical environments. Implementation of EBP facilitates effective clinical decision making and superior patient outcomes through the integration of the best available evidence, expert opinions, and patient preferences (Melnyk, Fineout-Overholt, Gallagher-Ford, & Kaplan, 2012). Therefore, EBP competency is an essential requirement for healthcare professionals (Tilson et al., 2011).

Developing EBP competency in nurses requires their acquiring competencies in areas such as informatics literacy, research methodology, and statistics to provide a requisite foundation for further learning. Therefore, cultivating EBP competency rarely achieves remarkable results after only a few courses or a short-term continuing education program (Melnyk & Fineout-Overholt, 2014). Nursing scholars have proposed that EBP teaching should be introduced early in undergraduate nursing education and EBP concepts should be integrated throughout the undergraduate nursing curriculum to connect multiple levels of prerequisite knowledge to equip undergraduate nursing students with the EBP competencies necessary to satisfy future practice needs (Melnyk, 2013; Nickerson & Thurkettle, 2013).

In response, a number of EBP curricula have been proposed in Western countries. However, shortcomings in these curricula include the integration of EBP concepts into limited aspects of the nursing curriculum (Bloom, Olinzock, Radjenovic, & Trice, 2013; Finotto, Carpanoni, Turroni, Camellini, & Mecugni, 2013), unresolved uncertainties regarding the applicability/adequacy of these curricula in actual teaching contexts (Rolloff, 2010), and ambiguities regarding how to integrate EBP content into professional nursing courses (Finotto et al., 2013). In addition, these curricula expect that undergraduate students will attain desired competencies in critical appraisal and the synthesis of research evidence by their third year (Bloom et al., 2013; Rolloff, 2010). On the basis of the abovementioned uncertainties and the different cultural contexts, it is uncertain whether these curricula would be appropriate for teaching nursing students in Taiwan.

In Taiwan, EBP is not only strongly emphasized in clinical practice but also valued in nursing academia (Mu, Tsay, & Chang, 2013). However, a national survey revealed that EBP teaching has been primarily conducted either independently or as one or two stand-alone lectures during nursing courses, with the teaching of clinical applications of evidence identified as the major challenge in EBP teaching (Hung, Huang, Tsai, & Chang, 2015). Therefore, developing a curriculum that integrates EBP concepts and teaches students how to apply evidence in clinical practice is an urgent issue in Taiwan.

In addition to selecting the essential EBP knowledge and skills for inclusion in undergraduate nursing curricula, educators must consider effective strategies to enhance the affective domain learning of EBP to foster a positive attitude toward EBP in students (Ryan, 2016). Generally, the steps for EBP, including search for evidence, formulation of answerable questions, and the critical appraisal and integration of available evidence, are considered to be essential EBP knowledge and skills that students must learn and master (Levin & Feldman, 2012). Several studies have shown that, although classroom teaching improves their EBP knowledge and skills, students should also enrich their EBP experience in clinical settings to enhance their attitudes toward EBP and their confidence in implementing EBP (Brown, Kim, Stichler, & Fields, 2010; Zhang, Zeng, Chen, & Li, 2012). However, the creation of a positive EBP learning experience in a clinical setting for students may be challenging. This is because transforming evidence into practice involves multiple complex factors such as the readiness of students and nurses as well as organizational readiness (Coomarasamy & Khan, 2004; Saunders & Vehviläinen-Julkunen, 2016; Solomons & Spross, 2011), which may increase the difficulties in enabling students to experience the implementation of EBP in clinical settings. Simulation-based teaching may offer a solution for surmounting these obstacles to EBP teaching. Simulation-based teaching has been reported as an effective teaching method in nursing education (Cant & Cooper, 2010) in terms of improving critical thinking ability and self-confidence in nurses (Alamrani, Alammar, Alqahtani, & Salem, 2018). In EBP teaching, Meeker, Jones, and Flanagan (2008) found that simulating clinical patient care scenarios enables students with limited clinical experience to practice formulating clinical problems and developing feasible solutions. Therefore, simulated patient care scenarios may be a useful teaching strategy for students to learn EBP skills and practice implementing EBP.

Therefore, the purpose of this study was to construct an EBP curriculum by systematically integrating EBP essential knowledge and skills into a 4-year undergraduate nursing curriculum and developing clinical scenarios to support EBP teaching.

## Methods

### Design

Action research (AR) has been shown as an effective approach to improve teaching quality and solve curriculum problems, with teachers applying self-reflection to identify problems and develop solutions that improve their education practice (Kemmis, McTaggart, & Nixon, 2014). Accordingly, AR was applied to develop and evaluate the undergraduate EBP nursing curriculum in this study.

AR usually involves an iterative cycle that starts from the identification of initial problems and then works to formulate possible solutions (planning), take appropriate actions (action), and reflect on the process to further plan and guide the iteration process in the next cycle (observation and reflection; Kemmis et al., 2014) to help participants improve their practice. In following this approach, the first cycles of this AR study started by identifying the need to construct a nursing curriculum that integrates EBP concepts. Subsequent iterative cycles proceeded forward based on the reflections of the participants in the prior cycle. Three iterative AR cycles were conducted in a department of nursing at a research-based university in southern Taiwan from July 2011 to August 2014, with approval from the institutional review board (A-ER-101-127).

### Setting

This study was carried out in a department of nursing that offers a 4-year baccalaureate nursing program and enrolls approximately 40 students each year. One hundred twenty-eight credits, including professional nursing and general education credits as well as 1,016 hours of clinical practicum, are required to earn a bachelor of nursing degree. Nineteen educators worked in this department. All of these educators had EBP teaching experience in clinical settings, and 12 had received EBP training from the Taiwan Joanna Briggs Institute Collaborating Center.

### Participants

Eleven nursing teachers with EBP teaching experience, 68 third- and fourth-year undergraduate students with EBP learning experience, and three external EBP education experts with Joanna Briggs Institute “Train the Trainer” certification participated in this study.

### Data Collection

AR may require that qualitative and quantitative data complement each other to provide comprehensive information about what to do and how to proceed through the AR cycles (Bryman, 2012). Accordingly, this study collected information using focus group interviews, external course-review documents, and questionnaire surveys.

The focus group interview was the main method used to inform and guide the three AR cycles in this study. During the three AR cycles, focus group interviews were held repeatedly to collect the observations and reflections of participants. These focus groups were moderated by the chief researcher (Y. J. Chang), who was also a teacher in the department of nursing. All of the group meetings were audio-recorded and then transcribed verbatim for use in the analysis.

Self-developed, external course-review documents were applied in the first cycle that comprised items reflecting the design of the preliminary EBP curriculum. These documents, including learning objectives, learning outcomes, teaching content, and learning activities, were mailed to the external experts with a request that they judge the completeness and appropriateness of the curriculum. Completeness was defined as an EBP curriculum that contained the overall concepts of EBP, whereas appropriateness indicated whether the design of the teaching content and learning activities matched expected learning outcomes and learning objectives. The experts were asked to rate each item in the documents on a 4-point scale, with 1 = *very inappropriate* and 4 = *very appropriate*.

The “EBP Attitude Questionnaire,” applied in Cycle 3, was adopted from parts of the EBP Knowledge, Attitude, and Behavior Questionnaire that was developed previously to assess the EBP knowledge, attitudes, and behaviors of nurses after participating in an EBP training program. The content validity index of this questionnaire is .86, whereas the internal consistency earned a Cronbach α of .74 (Lee, Wang, & Chang, 2011). This questionnaire consists of 10 items that are scored using a 4-point Likert scale, with 1 = *strong disagreement* and 4 = *strong agreement*. The total possible score range is 10–40, with higher scores indicating more positive attitudes toward EBP.

### Action Research Process

In response to the American Association of Colleges of Nursing (2008) appeal that baccalaureate nursing students should acquire sufficient EBP competency before they graduate, the participating department of nursing determined that applying empirical scientific strategies to explore, answer, and resolve clinical practice problems should be a core competency of undergraduate students. However, before this study, relevant EBP elements were only taught in certain courses. Therefore, the first cycle of AR in this study (planning stage) began after identifying the need to develop an EBP nursing curriculum. In this cycle (July 2011–August 2012), six EBP-experienced teachers in charge of core nursing courses were grouped by the chief researcher. In the group, the chief researcher used a series of questions regarding EBP curriculum design to encourage the participating teachers to discuss and share their recommendations. Then, focus groups were held repeatedly to confirm these recommendations and to continue discussing other elements of the EBP curriculum not yet covered or agreed upon (action stage). Three focus group interviews were held in the first cycle. After framing the preliminary EBP curriculum, three external EBP education experts validated this curriculum using the external course-review documents (observation and reflection stage).

The second cycle (August 2012–July 2013) began after obtaining the reflections of the participants on the first cycle, with the goal of evaluating the feasibility of the preliminary EBP curriculum in an actual teaching context (planning stage). Four teachers who were responsible for teaching core nursing courses, four teachers in charge of students' clinical practicum, and 32 third- and fourth-year students participated in this cycle. Because the EBP course was not a compulsory course at the time, before implementation of the preliminary EBP curriculum, a coordination meeting was held with the participating teachers to discuss how to implement the related teaching content and learning activities. Afterward, the preliminary EBP curriculum was implemented for two consecutive semesters (action stage). At the end of each semester, focus groups were held to characterize the experiences of the teachers and students for the purpose of further modifying the EBP curriculum. Overall, two teacher focus groups and two student focus groups were conducted during this cycle (observation and reflection stage).

**Figure 1.**
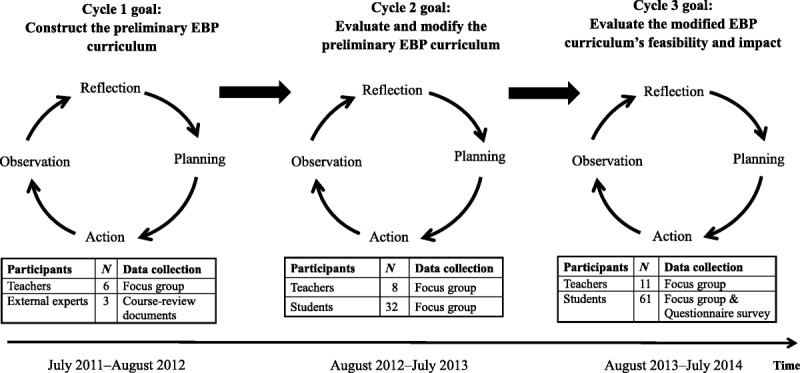
The process used to develop the undergraduate evidence-based practice (EBP) curriculum.

The third cycle commenced (August 2013–July 2014) after the preliminary EBP curriculum was modified based on the reflections of participants in the second cycle. This cycle included three tasks: (a) implement the entire modified EBP curriculum, (b) continually assess the feasibility of the modified EBP curriculum, and (c) evaluate the impact of the EBP curriculum on the EBP competency and attitudes of students toward EBP. Before implementation, the modified EBP curriculum was submitted to the nursing department's curriculum committee and presented at the departmental meeting for approval. After obtaining support from the dean and the committee, the researchers communicated with the teachers in charge of each designated course to characterize their needs for EBP teaching and share their experiences from the previous cycles of EBP teaching (planning stage). Subsequently, the modified EBP curriculum was implemented for two consecutive semesters (action stage). At the end of each semester, focus groups were held to evaluate the feasibility of the curriculum, and the EBP attitude questionnaire was used to assess the impact of the EBP curriculum on student attitudes. Eleven teachers and 61 third- and fourth-year students participated in this cycle. Two teacher focus groups and two student focus groups were held during this cycle (observation and reflection stage). The goal of each cycle, the data collection methods used, and information on the participants are outlined in Figure 1.

### Data Analysis

The verbatim transcripts were analyzed using deductive qualitative content analysis based on the categories that previous studies had formulated or on theories designed to code the transcript (Hsieh & Shannon, 2005; Vaismoradi, Turunen, & Bondas, 2013). Thus, relevant elements of curriculum design were used as schemes to code the transcripts.

The external course-review documents were analyzed using the concept of content validity index (Polit, Beck, & Owen, 2007). The content of items that received either a score of 3 or 4 from the experts on a 4-point scale in the external course-review documents was deemed to be appropriate. All of the items were rated as appropriate by the three external experts in this study.

The questionnaire survey regarding student EBP attitudes in the third cycle was analyzed using a paired *t* test, with significant differences between the pretest and posttest scores set at *p* < .05.

## Results

### Cycle 1: Preliminary Evidence-Based Practice Curriculum Construction and Simulated Scenario Development

The participating teachers considered that EBP education should be conducted along with instruction in relevant background knowledge. Accordingly, three levels of learning objectives with corresponding learning outcomes, teaching content, and learning activities were developed and embedded into the core nursing courses for students of different academic years. The overall preliminary curriculum framework is shown in Tables 1 and 2.

**TABLE 1.**
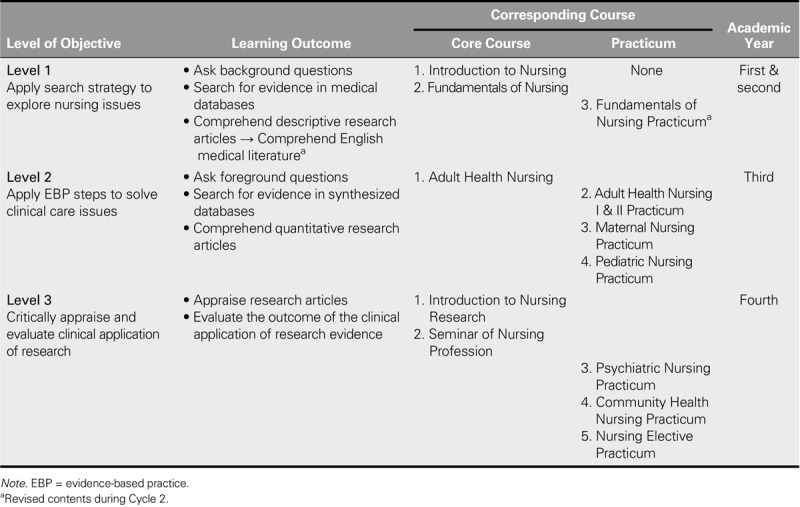
Learning Objectives and Learning Outcomes of the Developed EBP Curriculum

**TABLE 2.**
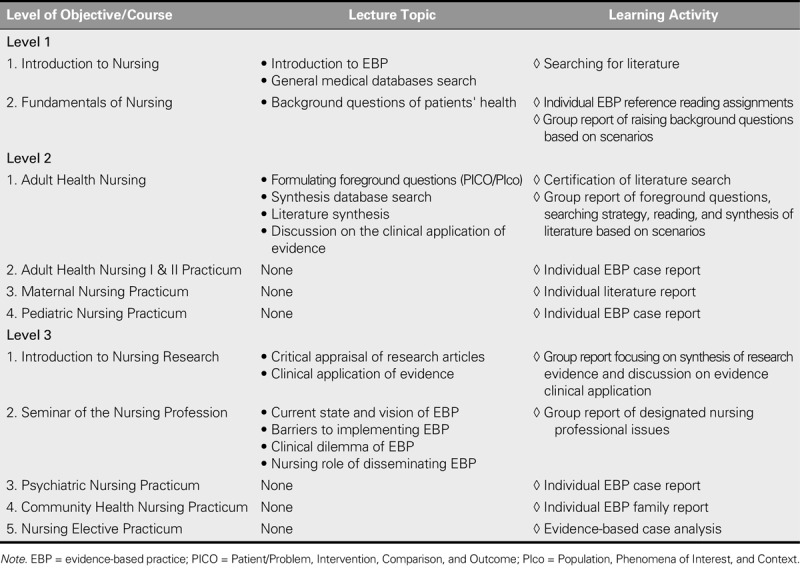
Preliminary Lecture Topics and Learning Activities of the Developed EBP Curriculum

#### Learning objectives and learning outcomes of each level

As shown in Table 1, as the first- and second-year students were beginners in the nursing field and only starting to acquire basic knowledge related to nursing, biomedical, and behavioral sciences, Level 1 teaching focused on developing the ability of students to ask questions and acquire information literacy and literature comprehension to support subsequent EBP learning. After students had acquired nursing knowledge and begun to synthesize learned knowledge and skills related to solving clinical care problems during their third year, Level 2 teaching advanced the abilities of students to search in synthesized databases and integrate evidence to address clinical care problems. When the students entered their last year and began acquiring knowledge related to research methodology, they were guided to analyze and identify the obstructions to nursing professionalism. Therefore, Level 3 teaching focused on critical evidence appraisal and on encouraging students to reflect on the outcomes of research evidence. The expected learning outcomes were set according to the learning objectives of each level (Table 1).

#### Teaching content and core courses at each level

To help students achieve the learning objectives and outcomes listed in Table 1, corresponding teaching content was developed and core nursing courses were integrated based on their original teaching elements to deliver the EBP teaching content appropriately for each level (Table 2).

Introduction to Nursing and Fundamentals of Nursing were designated as the two core courses for Level l. Because Introduction to Nursing is the first course to help students gain insight into the nursing profession and to teach elements of credible source searches, this course was designed to introduce the basic concepts of EBP and to teach students how to perform a general database search. In addition, the nursing processes taught in Fundamentals of Nursing, which emphasize the integration of knowledge of basic medicine, physical assessment, and human development as a strategy for identifying specific patient care problems, echoed elements of the background-question-raising procedure introduced in the EBP steps. Therefore, the teaching content of the nursing processes in this course was modified to emphasize the background questions associated with patient care issues.

Adult Health Nursing was designated as the core course for Level 2 because the objectives of this course help enable students to apply the nursing process to identify patient problems and further develop evidence-based interventions that satisfy patient needs, which are relevant to the expected learning objectives of Level 2. In addition, because the understanding of the experiences and values of patients is also emphasized in our curriculum, the lecture content of this course was further enhanced to include quantitative and qualitative types of foreground question formulation, a synthesized database search for identifying quantitative and qualitative evidence, and the integration of evidence.

Introduction to Nursing Research and Seminar of the Nursing Profession were designated as the two core courses for Level 3. Introduction to Nursing Research aims to increase student competence in evaluating research articles and their clinical application, whereas Seminar of the Nursing Profession aims to guide students to discuss nursing professionalism and critically analyze the factors affecting the development of the nursing profession and the practice of nursing. These teaching objectives are relevant to the expected learning objectives of Level 3. Thus, Introduction to Nursing Research was designed to integrate the previously learned EBP knowledge and to teach the appraisal of research evidence and application. Finally, the issues related to the vision of EBP, clinical dilemmas of EBP, and the role of nurses in disseminating EBP were added to the lectures of the Seminar of the Nursing Profession.

#### Learning activities at each level

Various individual and group learning activities were developed for classroom and practicum courses to provide students with opportunities to practice EBP content (Table 2). In particular, assignments consisting of clinical care scenarios and EBP processes were developed to familiarize students with the EBP steps. The scenario assignments were developed according to the concept of critically appraised topics (Sadigh, Parker, Kelly, & Cronin, 2012) and commenced with a simulated nursing scenario followed by instructions that were designed to guide students to think about the scenarios and complete each EBP step. The simulated nursing scenarios were developed preliminarily by the research team and then reviewed and modified by the participating teachers.

In addition, the original care-report assignments of the practicum were modified to emphasize the identification of nursing problems, the development of evidence-based interventions, and the application of interventions consistent with evidence and patient preferences.

#### Initial evaluation and other reflections

After establishing the preliminary EBP curriculum, three external EBP experts were invited to verify its completeness and appropriateness. Although the external experts approved the preliminary EBP curriculum, they recommended that the feasibility of the three-level learning scheme be assessed through actual implementation in lectures and practicums.

Moreover, in addition to providing perspectives for constructing the concrete and visible curriculum framework, the participating teachers, on the basis of their experience, reported that guiding students to form answerable questions was the biggest challenge in EBP teaching. The reason behind this difficulty may be that Taiwanese students are accustomed to learning passively and receiving what is taught. Therefore, creating a learning climate in which students are encouraged to ask questions and learn actively was important to the successful implementation of the EBP curriculum.

### Cycle 2: Evaluation and Modification of the Preliminary Evidence-Based Practice Curriculum

After the preliminary EBP curriculum was implemented for two consecutive semesters, the teachers and students agreed with the learning objectives of the curriculum. However, some learning outcomes, teaching content, and learning activities required further modification, and certain teaching elements needed to be added. These modifications are summarized in Tables 1 and 3.

**TABLE 3.**
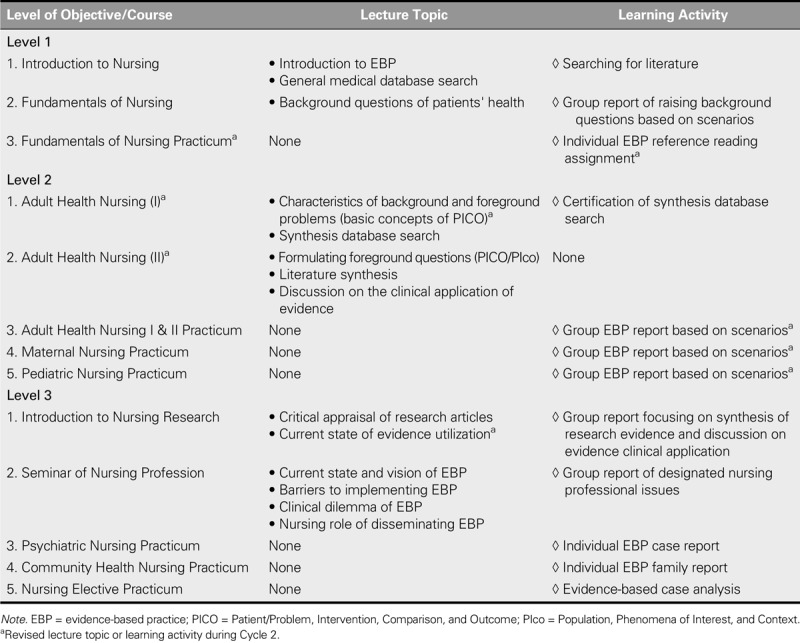
Modified Lecture Topics and Learning Activities of the Developed EBP Curriculum

#### The modification of learning outcomes for Level 1

The teachers reported that they preferred guiding students to read appropriate literature according to clinical issues pertinent to their own interests rather than confining them to reading a preassigned type of research article. Therefore, the learning outcomes for Level 1 were revised (Table 1).

#### The modification of teaching content for Levels 2 and 3

By evaluating the performance of students on the scenario assignments, the teachers found that, despite the classroom lectures, students still had difficulty formulating foreground questions. A possible reason for this difficulty was that students were not equipped with sufficient background knowledge, which caused them to ask background questions related to patient problems rather than foreground questions. Therefore, the teachers concluded that Level 2 teaching should be conducted in two phases to simultaneously build the background knowledge of students as a foundation for formulating foreground questions while strengthening the understanding of students of different types of clinical problems. Furthermore, because the application of evidence to practice could not be directly experienced in Introduction to Nursing Research, the course content was also revised to include discussions of the current state of evidence utilization in clinical settings (Table 3).

#### The modification of learning activities

Because the third-year students had limited EBP knowledge, the teachers decided that the learning activities of Level 2 should all be handled as group work to facilitate better learning outcomes. In addition, the teachers concluded that the scenario assignments should also be handled as learning activities for the clinical practicum. One reason for this change was that time was limited for students to practice the EBP scenario assignments in the classroom. Another reason was that the design of the scenario assignments was based on common clinical patient care problems, and the teachers thought that practicing the scenario assignments in a clinical setting would give students better opportunities to correlate these scenarios with actual patient care and to observe the application of EBP. Other modifications of the learning activities are shown in Table 3.

#### Other reflections

The teachers perceived that EBP teaching in this stage of nursing education was challenging and time consuming, especially when guiding students through each EBP step in the scenario assignments. To support future EBP teaching, teachers suggested that graduate-level teaching assistants could help students search, understand, and integrate research evidence. In addition, self-learning supplementary materials consisting of examples on the differences between background and foreground questions, formulating PICO/PIco (Patient/Problem, Intervention, Comparison, and Outcome, or Population, Phenomena of Interest, and Context) frameworks, learning search strategies, understanding levels of evidence and resources, applying various research methodologies, and providing guidance for reading various types of research articles should be developed and shared on a digital teaching platform to help students complete additional self-learning exercises whenever necessary. Another important suggestion proposed by the participating teachers was to better engage the EBP learning motivation of students. To this point, integrating EBP concepts into events that students are likely to encounter in their lives may be an effective approach.

### Cycle 3: Evaluation of the Feasibility and Impact of the Modified Evidence-Based Practice Curriculum

After the modified EBP curriculum was implemented comprehensively for two consecutive semesters, the teachers and students generally approved of the modified EBP curriculum. By evaluating the progress of students through the learning activities and designated assignments, the teachers concluded that the students were able to achieve most of the expected learning outcomes. Moreover, the students were satisfied with their own performance, especially with regard to reviewing and integrating literature, and also expressed that the EBP learning experiences raised their level of inquiry and truth-seeking motivation and improved their confidence in clinical settings. Furthermore, the pretest and posttest questionnaires (Table 4) that were completed by 57 students revealed that the students earned significantly higher posttest scores after receiving the EBP education, which indicates that they had a more positive attitude toward EBP than before they completed the courses (*p* < .004).

**TABLE 4.**
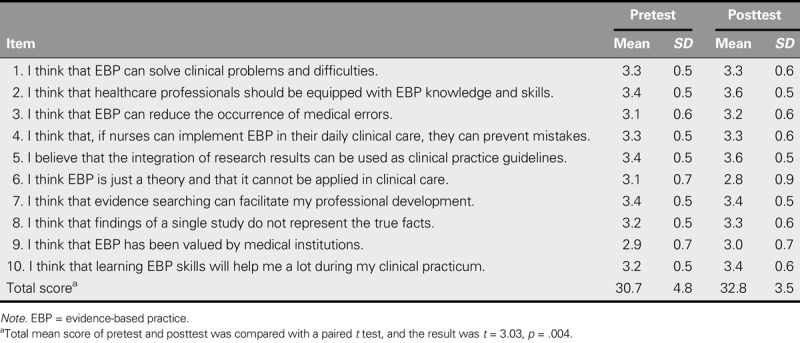
Impact of the Developed EBP Curriculum on the EBP Attitudes of Students (*N* = 57)

#### Other reflections

Although the teachers were satisfied with the performance of the students, they believed that some effort should be made toward improving the students' insufficient understandings of foreground and background questions as well as their weaknesses in interpreting statistical results in research articles, using synthesized databases, and appraising and applying evidence. Therefore, clarifying the definition of foreground and background questions, providing reliable resources for answering foreground and background questions, and enhancing students' comprehension of the application of critical appraisal tools and holistic thinking about the risks, ethics, and safety issues involved in using evidence were proposed for use as teaching elements of EBP education. Furthermore, including strategies for interpreting the statistical results of research articles in biostatistics courses and for repetitive practice in searching the databases in other nursing courses was also suggested.

The participating teachers further suggested that clinically answerable questions (PICO/PIco format) that had been formulated and processed could be used as effective teaching resources to save time when guiding students to form answerable questions and to identify appropriate literature for helping students learn subsequent EBP steps. In addition, other support resources such as faculty development and additional teaching manpower should be engaged to more fully promote implementation of the EBP curriculum.

## Discussion

A systematic EBP undergraduate nursing curriculum with three levels of learning objectives and corresponding learning outcomes, teaching content, and learning activities was developed. Our EBP curriculum was organized in a vertical structure from basic to advanced, simple to complex, and group cooperative learning to individual independent learning to build the EBP knowledge and competency of students progressively. Furthermore, interprofessional nursing courses were integrated in a horizontal structure to collaboratively assist students to achieve the learning objective of each level. This curriculum was designed not only to deliver relevant EBP knowledge but also to coordinate with various learning activities (the clinical scenario assignments, in particular) to deepen student comprehension of the EBP process and consolidate their EBP knowledge. A preliminary evaluation found that this curriculum successfully fostered the EBP competency of students, increased their confidence in clinical practice, and promoted their adoption of a positive attitude toward EBP.

Possible reasons for the effectiveness of the developed curriculum are that its design emphasized that EBP teaching be conducted in parallel with the accumulation of background knowledge (Nickerson & Thurkettle, 2013) and that it incorporated multiple pedagogical strategies, including lectures, group collaboration, and linking academic learning with clinical practice, in an effort to maximize EBP learning (Christie, Hamill, & Power, 2012). Furthermore, the findings of this study suggest that allowing students to practice EBP scenario assignments during the clinical practicum may greatly and positively impact their attitudes toward EBP. The reason for this effect is that working on these assignments during the clinical practicum not only provides students with opportunities to learn and experience how to judge the feasibility of available evidence in clinical practice through faculty guidance and role modeling but also enables students to visualize the impact of EBP on clinical care. These learning experiences have been shown to improve student attitudes toward EBP (Brown et al., 2010). Moreover, as undergraduate nursing students should be expected to be users of evidence in future clinical practice settings, the teaching elements addressing the clinical application of evidence such as critical appraisal instruments that guide students in analyzing the clinical applicability of research evidence (Nadelson & Nadelson, 2014) and synthesized databases that allow students to instantly obtain the preappraised and integrated evidence (Ahmadi, Baradaran, & Ahmadi, 2015) were also emphasized in this curriculum. These efforts may enable students to better experience the feasibility and importance of EBP and lead to a more positive attitude toward EBP and greater confidence in practicing EBP in the clinical practicum.

Nevertheless, our teachers stated that EBP teaching was still replete with challenges that require multiple strategies and sufficient investments of resources. Although most teachers in this study had received EBP training, teachers still strongly perceived a need for advanced teaching skills and knowledge. A possible explanation for this finding is that, despite the growing emphasis in the literature on the importance of developing the EBP competency of nursing students, relatively little attention has been paid to developing the knowledge and skills of faculty that will be necessary to integrate EBP concepts into nursing courses and clinical practicums and to teach EBP more effectively (Levin, 2014). Workshops, small group activities, short courses, peer mentoring, and online resources are proposed as feasible strategies for faculty to acquire and exchange effective EBP teaching strategies (Snell, 2014). These methods could be considered in future work to enhance the EBP teaching competency of faculty.

Furthermore, adequate resources are indispensable to curriculum implementation (Iwasiw & Goldenberg, 2014). Despite the initial idea of the researchers of this study to integrate existing teaching elements of the original professional courses into the teaching of EBP to lessen the burden on teachers, the participating teachers still expressed the need for more resources to support their teaching. Establishing interprofessional teaching partnerships (such as collaboration with librarians to better teach on the use electronic databases and with clinical professionals to better teach on the application of evidence) may be considered as potentially supportive EBP teaching resources (Levin & Feldman, 2012).

Finally, this study identified showing effective leadership as important for constructing and implementing a new curriculum (Long et al., 2014) because related preparatory work had already begun on the construction of this EBP curriculum. In the present case, a faculty member (Y. J. Chang), who had received in-depth EBP training and had administrative experience, led the curriculum team. The contributions of this leader included seeking funding from the institution to support EBP training for faculty members, participating in the program committees to promote a consensus among teachers to set EBP as a core competency for nursing students, and convening the teacher groups that were responsible to design the EBP curriculum. During curriculum implementation, this project leader worked continuously with the faculty as a mentor and shared knowledge about EBP teaching. This leadership was, in this study, and will continue to be a key factor in constructing and implementing an effective EBP curriculum.

### Limitations

As a preliminary study, this study created the framework for a systematic EBP curriculum and tested its feasibility and effectiveness on the EBP competency of students, mainly using subjective methods. This evaluation approach may not be sufficient to reflect the viability or effectiveness of a specific curriculum in practice (Iwasiw & Goldenberg, 2014). Therefore, we suggest that a future study be conducted to combine objective and subjective methods to continuously assess the appropriateness of the EBP curriculum and evaluate its impact on multiple domains of the EBP competency of students.

### Conclusions

By integrating EBP concepts into a 4-year baccalaureate nursing program, this study developed a systematic and effective EBP nursing curriculum with valuable pedagogical content and strategies. The developed curriculum successfully increased the competency and confidence of the students, which positively impacted their attitudes toward EBP. The process of developing and implementing the EBP curriculum and the resulting information generated may provide a valuable reference for other nursing schools.
